# Algae Supplementation for Exercise Performance: Current Perspectives and Future Directions for Spirulina and Chlorella

**DOI:** 10.3389/fnut.2022.865741

**Published:** 2022-03-07

**Authors:** Tom Gurney, Owen Spendiff

**Affiliations:** School of Life Sciences, Kingston University, Kingston upon Thames, United Kingdom

**Keywords:** algae, spirulina, chlorella, ergogenic aids, antioxidants, exercise performance

## Abstract

Nutritional clinical trials have reported algae such as spirulina and chlorella to have the capability to improve cardiovascular risk factors, anemia, immune function, and arterial stiffness. With positive results being reported in clinical trials, researchers are investigating the potential for algae as an ergogenic aid for athletes. Initial studies found spirulina and chlorella supplementation to increase peak oxygen uptake and time to exhaustion, with the mechanistic focus on the antioxidant capabilities of both algae. However, a number of oxidative stress biomarkers reported in these studies are now considered to lack robustness and have consequently provided equivocal results. Considering the nutrient complexity and density of these commonly found edible algae, there is a need for research to widen the scope of investigation. Most recently algae supplementation has demonstrated ergogenic potential during submaximal and repeated sprint cycling, yet a confirmed primary mechanism behind these improvements is still unclear. In this paper we discuss current algae supplementation studies and purported effects on performance, critically examine the antioxidant and ergogenic differing perspectives, and outline future directions.

## Introduction

While much attention and scientific interest surrounding the potential for algae as a functional food has been gaining momentum in the past two decades, written reports of its consumption in human diets go as far back as 300 A.D. in China (1). It is apparent that the high nutritional value of algae has long been considered but it is only recently that various bioactive compounds and nutrients have been detected in both seawater and freshwater algae (2). Chlorella and spirulina are currently the two most well-established algae supplements, with mass worldwide cultivation and production starting in the early 1940s, primarily due to both algae comprising an abundant breadth of micronutrients that may promote human health (2, 3). Indeed, reports suggest both types of algae may improve cardiovascular risk factors (4), anemia (5), immune function (6), and arterial stiffness (7). Recent investigations have therefore attempted to bridge the gap between these positive clinical reports and the applicability/potential of algae supplementation for use in exercise performance as an ergogenic aid.

The nutritional composition of spirulina and chlorella makes them particularly attractive to biopharmaceutical and nutraceutical industries. Both contain high levels of protein (up to 65% dry weight), which include all the essential and non-essential amino acids (1), making them a plausible additional supplement to consider for vegetarians and vegans. Indeed, early observations reported tribes alongside Lake Chad consuming spirulina as a substitute for meat (8). Fatty acids, pigments, vitamins, and minerals are also in their abundance in both algae (1–3, 9), meaning spirulina and chlorella can be a viable high nutrient source for human consumption. For a thorough nutritional analysis breakdown, the authors refer the reader to Andrade and colleagues review (9).

Despite spirulina and chlorella possessing similar nutritional qualities, it is important to note that there is a key difference in their production process. Spirulina, a filamentous cyanobacterium, lacks a cellulose cell wall (3) meaning that once harvested and dried it is ready for human consumption immediately. This not only makes the production process shorter but consequently makes spirulina more attractive and cheaper to mass cultivate. Additionally, the lack of a cellulose cell wall means the digestibility and bioavailability of spirulina is high (3). In contrast, chlorella in its natural form contains a cellulose cell wall, which must be mechanically broken-down during production before being ready for human consumption (2). A complex, delicate, and expensive step that is essential for safe human use. This key difference in the production process may explain the disparity in the current research output volume of spirulina and chlorella within Sport and Exercise Nutrition research.

To date, due to the nutrient complexity and density in both algae, there appears to be a distinct lack of consensus on what primary mechanism may cause the efficacious results following supplementation for exercise performance. For instance, the phycocyanin, β-Carotene, cysteine and vitamin content in spirulina led to initial investigations comparing oxidative stress biomarkers after exercise (10–13). However, lately there has been a shift in focus for which constituents in spirulina and chlorella might be the mechanism of action for when improvements in exercise performance parameters are observed (14–17). Is it apparent that the nutrient density of these commonly found algae have potential as an effective nutritional supplement for both health and performance in athletes around the world, yet research is still equivocal. Therefore, the aim of this manuscript is twofold: (1) to evaluate current perspectives in algae supplementation for exercise performance, and (2) to explore and suggest possible future directions.

## Antioxidant Approach

It was previously a widespread acceptance that antioxidant supplementation can combat possible human redox imbalance *via* the scavenging of reactive oxygen species (ROS) and reactive nitrogen species (RONS) that are naturally produced by the mitochondria during exercise (18). Principally, the balance between oxidants and antioxidants (human redox status) is dependent on intricate signaling pathways, which can be altered and influenced by exogenous antioxidant supplementation. Various oxidative stress biomarkers are also associated with the multidimensional antioxidant defense system, which if improved may suggest human redox status maintenance (18). Alleviating skeletal muscle damage and fatigue, including the improvement in recovery, are thought to be induced by antioxidant supplementation *via* the balance of human redox status. As such, much attention on how antioxidants derived from algae supplementation may benefit exercise performance has stemmed from early *in vitro* reports demonstrating algae exerting high antioxidant enzyme and radical scavenging activity (19–21), possibly *via* the activation of the NRF2 signaling pathway (22), including the prevention of lipid peroxidation (19). Similarly, these findings appear to be consistent in rodent studies whereby oxidative stress markers such as Malondialdehyde (MDA), total antioxidant capacity (TAC), and lipid peroxidation improve following spirulina or chlorella supplementation (23, 24).

Spirulina has received the most attention in humans; previous authors have reported spirulina to improve time to exhaustion whilst running (10, 11) and V̇O_2max_ whilst cycling (13). Another report demonstrated spirulina supplementation to improve recovery heart rate and peak lactate following a cycling V̇O_2max_ (12). The consensus of the findings indicate that spirulina possesses antioxidant mechanistic potential during/after exercise, as demonstrated by an improvement in a variety of oxidative stress markers [MDA, thiobarbituric acid-reactive substances (TBARS), TAC, lipid peroxidation and reduced glutathione (GSH)] (10–12). It was postulated that these biomarker improvements were an indication of spirulina exhibiting ROS/RONS scavenging potential which therefore may have alleviated the oxidative stress associated with such exercise intensities. In clinical trials, a meta-analysis also concluded that spirulina has positive marginal effects on TAC and superoxide dismutase capacity (25). Conflicting evidence does, however, suggest that spirulina has no effect on similar oxidative stress biomarkers and exercise performance (26, 27). As for chlorella, rodent studies have demonstrated the capability of chlorella to influence similar oxidative stress biomarkers (28, 29). However, in exercise performance human studies and in contrast to spirulina, other constituents found in chlorella have been suggested to be causing the ergogenic effect which we discuss later. Nevertheless, evidence that algae supplementation influences human redox status is equivocal, particularly when the direct links between antioxidant changes and improvements in exercise tolerance/performance are not clear. For example, several of the key oxidative stress biomarkers used in the aforementioned studies are now considered to lack robustness (30). Specifically, the flawed assays of TBARS and TAC are inadequate today in redox biology (30). Considering spirulina contains approximately 0.45 g of cysteine/100 g and that adequate cysteine levels are essential for optimal glutathione concentration levels, redox status, and muscle function (31), equivocal results might also be attributed to the inter-individual baseline differences of glutathione before starting supplementation. For example, a recent study demonstrated that individuals with moderate-high baseline levels of glutathione did not benefit from N-acetylcysteine supplementation, yet those with low baseline levels improved V̇O_2max_, time trial and Wingate tests by 13.6, 15.4, and 11.4%, respectively (32).

It is apparent that algae contain a wide range of constituents capable of free radical scavenging *in vitro*, yet a well-characterized antioxidant action of a molecule does not necessarily translate into complex *in vivo* biology, especially in exercise performance (33). As a consequence, there remains a considerable knowledge gap in the antioxidant efficacy of algae supplementation on exercise performance in humans. Indeed, antioxidant supplementation for adjusting redox balance during/after exercise is somewhat controversial as supplementation may blunt key favorable endurance and high-intensity training adaptations *via* the suppression of key cell signaling pathways associated with mitochondrial biogenesis and hypertrophy (18, 34, 35). In fact, emerging evidence generally indicates that exercise-induced reactive species are essential for mediating the upstream of signals associated with the activation of redox-sensitive transcription factors (PCG-1α, HIF-1α, NF-κB, and NFE2L2) (18, 36). Such reactions are becoming increasingly more recognized as a fundamental element in fine-tuning human metabolism (36). Though further research is most certainly needed in chronic antioxidant supplementation studies, it is plausible to suggest that a net balance of intake is needed, including the careful consideration of supplementation timing. Considering the equivocal antioxidant research following algae supplementation, aside from some promising improvements in performance being observed, it is apparent that there is a need for research to consider different mechanisms of action. Indeed, the breadth of constituents found in algae mean that there is a vast amount of other possible mechanisms that can be investigated, this has become evident in a small amount of emerging research (14–17).

## Current Emerging Ergogenic Mechanistic Avenues Being Explored

Despite antioxidants being the major focus in algae supplementation for exercise performance, a few studies have offered differing perspectives on their constituents, and therefore mechanisms that might be the cause for the observed improvements in performance. Reports that spirulina can improve peak and average muscular force (15, 37) have led to one group of authors suggesting that spirulina’s high protein efficiency ratio (87%) and net protein ultilisation (92%) may have played an important role in achieving the positive results whilst administering alongside a training programme (37). Though this may well be a contributing factor, and despite roughly 65% of spirulina’s dry weight being a complete protein source, it is important to note that a substantial dosage of algae would have to be consumed to achieve the daily recommended protein intake.

There is also growing evidence that both spirulina and chlorella can induce vasodilatory effects. Spirulina may augment circulatory nitrate/nitrite whereby the phycocyanin constituent reportedly increases nitric oxide (NO) availability in the aorta and plasma in rats, including the increase in the expression of endothelial nitric oxide synthase (eNOS) (38, 39). Reviewing the ergogenic effects of nitrate on exercise performance are beyond the scope of this study but can be found elsewhere (40). However, this mechanism derived from spirulina supplementation and how it might influence exercise performance in humans has received little attention, despite the plausibility of spirulina possessing vasodilatory mechanistic properties in prior research (41, 42). To date, confirmation of increases in nitrate/nitrite following spirulina supplementation in humans has not been reported. Conversely, there is evidence that chlorella supplementation can induce increases in NO production in men (43). Indeed, a recent meta-analysis on chlorella supplementation and cardiovascular risk factors in clinical populations concluded that chlorella possesses antihypertensive properties (4). Even in healthy young and middle-aged men, chlorella has exhibited vasodilatory potential whereby reductions in arterial stiffness were observed following 6 g/day 28-day supplementation periods (7, 43). Employing the same dose and supplementation period, chlorella supplementation led to significant augmentations in peak oxygen uptake in young men (16, 17), with vasodilation being attributed to one of the possible mechanisms, though confirmation within this study was absent.

High bioavailable constituents found in chlorella such carotenoids (lutein, β-Carotene and zeaxanthin), polyunsaturated fatty acids (PUFA), linoleic acid and water-soluble fibers were also some of the purported mechanisms of action in the aforementioned studies, whereby it was suggested that these constituents bind to digested fat, increase plasma scavenging of LDL-C (low density lipoprotein), and reduce absorption of sterols (cholesterol) from the intestine (4, 44). Some speculation that the arginine found in chlorella was also conveyed to be a plausible mechanism of action (16, 17), though research into whether arginine may augment NO production and therefore positively affect aerobic exercise in healthy participants is controversial and conflicting (45). Further research is needed in both algae to confirm these possible vasodilatory effects and how this might impact exercise performance, yet the data emerging is promising.

An often overlooked yet plausible constituent found in algae that may contribute to positive ergogenic affects is iron. The good bodily assimilation and high bioavailability of iron from spirulina, primarily due to phytates and oxalates being absent, has resulted in several positive hemopoietic trends being reported in clinical populations (5, 46) and now in healthy sports men (14, 15, 47, 48). How this might influence exercise performance certainly deserves more attention but perhaps more importantly, the exact mechanism needs to be confirmed, particularly when spirulina supplementation causes increases in Hb in healthy sports men without a known iron deficiency. Nonetheless, it is well established that optimal iron stores are essential for daily hemoglobin (Hb) synthesis and that Hb plays a key role in the transportation of oxygenated oxygen from the lungs to the muscles (49). Previous authors have therefore suggested that spirulina may enhance oxidative capacity (12, 13, 41) and our latest work indicates improvements in Hb following spirulina supplementation may positively influence exercise performance in arm and leg cycling (14, 15). Some authors have also suggested that the possible nitrate concentration found in spirulina may stimulate the contraction of the spleen (a known dynamic reservoir of erythrocytes which can induce “spleen-related blood boosting”), speculation primarily due to their recent work which suggests 5 mmol nitrate supplementation can elevate circulatory Hb by up to 3% *via* splenic contraction (50).

## Limitations in the Field

Given the infancy of algae supplementation research in the Sport and Exercise Nutrition field, it is no surprise that an optimal dose/duration strategy are yet to be determined. Spirulina research has ranged from 1.5– 7.5 g/day to 7 – 60 days, whereas for chlorella there seems to be a little more uniformity. A handful of studies have employed 6 g/day for 3–4 weeks, though there is some promising research emerging that suggests acute single dosages are enough to elevate various biomarkers (44). Overall, this general lack of uniformity for dosages and supplementation duration is to be expected given the novelty and equivocal results so far in both algae.

It is apparent that there are some promising positive findings following either chlorella or spirulina supplementation, however, the mechanisms to any performance benefits are still not clear. Mechanistic confirmation is required to support such positive findings, particularly in the Sport and Exercise Nutrition field. Understandably, initial investigations are yet to pinpoint consistent mechanistic findings as both algae are complex in their nature, further work that focusses on a specific constituent is suggested. Despite this, previous studies should not be quickly disregarded as these preliminary investigations may prove to be key seminal work for future research.

## Future Directions and Conclusion

Algae are a diverse and complex species that deserve more attention in the Sport and Exercise Nutrition field and are also increasingly being used to mitigate elevated CO_2_ levels including the use in renewable energy, biopharmaceutical, and nutraceutical industries, particularly as we edge ever closer to a greener and more sustainable world (see Kahn et al. (51) for a review).

In the Sport and Exercise Nutrition field, further clarification is required on the efficacy of these algae when the nutritional and trained status of participants are taken into consideration. It is not yet established whether positive ergogenic effects might simply be due to improvements in deficiencies and/or nutritional status. For example, it is well understood that adequate nutritional status is key for optimal sport performance (52) and that the “one-size-fits all” approach is not appropriate due to individuals responding differently to the same nutrients and supplements (53). Personalized and tailored methods should therefore be considered to each individual. Throughout this manuscript we have highlighted several studies that show nutritional interventions are most likely to improve exercise performance outcomes when deficiencies are identified and corrected. Where possible, researchers should consider screening participants prior/during algae intervention trials. Athletes in the future might then be able to make informed decisions when considering if algae supplementation is necessary.

To the best of our knowledge, neither algae have been exclusively investigated with female athletes and considering spirulina’s high bioavailability of iron and the high prevalence of anemia in female athletes, spirulina may prove to be particularly effective in health and performance in the female athletic community.

Additionally, future research should also consider the quality of spirulina and chlorella powder. Spirulina and chlorella are packaged as “dry” biomass in powdered form which is consequently cold pressed into tablets or filled into capsules. The most common method of drying algae ready for human consumption is “spray drying” which involves exposing the algae to temperatures between 100 and 150 Degrees Celsius (54). However, it’s well established that the employing different drying strategies can influence the nutrient density of the algae (54). The freeze drying method for example has been suggested to be an ideal method for microalgae biomass due to the limited degradation of biomass nutrient quality (55). Further research should therefore consider comparing spray dried vs. freeze dried algae to assess any notable difference in bioavailability in humans.

Considering both the positive clinical and promising ergogenic aid reports, there needs to be a holistic approach to future studies, future work should consider how algae supplementation may not only influence health but also performance in athletes simultaneously. More mechanistic blood work needs to be conducted in this area to further support previous initial speculative research, nonetheless, the aforementioned studies highlighted in this report will form key foundations for algae supplementation research within the Sport and Exercise Nutrition field which will allow for future avenues to be explored. It is apparent that these algae possess many possible mechanisms of action, yet due to research being in its infancy, consistency in the research has been sporadic. We call for a more channeled and collaborative approach from researchers in their prospective fields, future collaboration among industries will provide a more comprehensive understanding in how best to ultilise algae for athletes around the world.

## Data Availability Statement

The original contributions presented in the study are included in the article/supplementary material, further inquiries can be directed to the corresponding author.

## Author Contributions

TG wrote and prepared the manuscript. OS prepared and revised the manuscript. Both authors approved final version of manuscript.

## Conflict of Interest

The authors declare that the research was conducted in the absence of any commercial or financial relationships that could be construed as a potential conflict of interest.

## Publisher’s Note

All claims expressed in this article are solely those of the authors and do not necessarily represent those of their affiliated organizations, or those of the publisher, the editors and the reviewers. Any product that may be evaluated in this article, or claim that may be made by its manufacturer, is not guaranteed or endorsed by the publisher.
